# Optimization of Iron Oxide Tracer Synthesis for Magnetic Particle Imaging

**DOI:** 10.3390/nano8040180

**Published:** 2018-03-21

**Authors:** Sabina Ziemian, Norbert Löwa, Olaf Kosch, Daniel Bajj, Frank Wiekhorst, Gunnar Schütz

**Affiliations:** 1 MR and CT Contrast Media Research, Bayer AG, D-13353 Berlin, Germany; gunnar.schuetz@bayer.com; 2 Physikalisch-Technische Bundesanstalt, D-10587 Berlin, Germany; Norbert.Loewa@ptb.de (N.L.); Olaf.Kosch@ptb.de (O.K.); Frank.Wiekhorst@ptb.de (F.W.); 3 University of British Columbia, Vancouver, BC V6T 1Z3, Canada; daniel.bajj@alumni.ubc.ca

**Keywords:** magnetic particle imaging (MPI), magnetic particle spectroscopy (MPS), iron oxide tracer, superparamagnetic nanoparticles

## Abstract

The optimization of iron oxide nanoparticles as tracers for magnetic particle imaging (MPI) alongside the development of data acquisition equipment and image reconstruction techniques is crucial for the required improvements in image resolution and sensitivity of MPI scanners. We present a large-scale water-based synthesis of multicore superparamagnetic iron oxide nanoparticles stabilized with dextran (MC-SPIONs). We also demonstrate the preparation of single core superparamagnetic iron oxide nanoparticles in organic media, subsequently coated with a poly(ethylene glycol) gallic acid polymer and phase transferred to water (SC-SPIONs). Our aim was to obtain long-term stable particles in aqueous media with high MPI performance. We found that the amplitude of the third harmonic measured by magnetic particle spectroscopy (MPS) at 10 mT is 2.3- and 5.8-fold higher than Resovist for the MC-SPIONs and SC-SPIONs, respectively, revealing excellent MPI potential as compared to other reported MPI tracer particle preparations. We show that the reconstructed MPI images of phantoms using optimized multicore and specifically single-core particles are superior to that of commercially available Resovist, which we utilize as a reference standard, as predicted by MPS.

## 1. Introduction

Magnetic particle imaging (MPI) is a new imaging modality, introduced by Gleich and Weizenecker in 2005, with the potential to enrich modern diagnostic imaging [1]. MPI technology is still under development [2], and significant challenges remain in streamlining the highly complex instrumentation as well as optimizing iron-oxide-based MPI tracers. The ideal tracer must have optimized physicochemical properties; a high magnetization, consistent morphology, and chemical stability in biological media are all necessary to generate high-quality three-dimensional images.

MPI has been suggested as being suitable for a wide range of applications, such as vascular diagnostic and interventional procedures, gastrointestinal and pulmonary imaging, as well as cellular and targeted imaging [3,4,5]. Further uses are envisaged with the development of multicolor MPI, implementing a signal separation acquired simultaneously from different tracer types or from tracers in different environments [6,7]. To date, only research scanners and a few preclinical demonstrators acquiring one-dimensional (1D), 2D, and 3D images are in use, aiming at establishing the relation between the tracer properties, scanner parameters, and the resulting sensitivity and spatial/temporal resolution of the image [8]. The feasibility of MPI for clinical imaging has yet to be demonstrated in a whole-body clinical scanner [9].

The first preclinical in vivo MPI experiments utilized Resovist (Bayer Schering Pharma, Berlin, Germany now available from I’rom Pharmaceutical Co Ltd., Tokyo, Japan), a formulation of superparamagnetic iron oxide nanoparticles and a clinically approved liver-specific magnetic resonance imaging (MRI) contrast agent. In the MPI community, Resovist has become a reference standard for performance measurements due to its exceptional signal stability and biocompatibility. The good MPI signal is explained by the presence of small elementary crystallites of 5 nm, forming compact aggregates of about 25 nm [10,11]. Since only a small fraction of the particles in Resovist contribute to the signal, further performance improvements of up to 2.5-fold are possible by size fractionation of this iron oxide tracer [12,13].

Aiming at higher MPI signal improvements, synthesis methods of iron oxide nanoparticles have been optimized by tailoring the properties of the superparamagnetic iron oxide nanoparticles (SPIONs). The particle size and local environment have an impact on the extent to which Néel and Brownian relaxation contributes to the overall magnetic relaxation, which is a key parameter for spatial resolution [14]. In addition to the iron core diameter and its environment, the relaxation mechanism is dependent on the frequency and the strength of the drive field [15]. Optimal iron oxide core diameter may vary for different drive field settings. The higher the frequency of the excitation field, the lower the optimum core diameter [16,17]. Another important parameter for SPIONs in MPI is the anisotropy of particles. A small core anisotropy and coercivity may enhance the performance of the MPI tracer [18]. When designing the tracer for use in vivo, care should be taken to optimize the surfactant coating to prevent the aggregation of particles and promote biocompatibility. The coating should be thin enough to minimize viscous relaxation, but sufficiently thick to prevent aggregation [19].

A common approach for obtaining improved MPI tracers is to purposely synthesize multicore clusters consisting of small iron oxide crystallites. A popular method is coprecipitation from ferrous and ferric chloride in aqueous media under basic conditions, in the presence of a stabilizing agent like dextran. Eberbeck et al. [20] reported on Nanomag-MIP NPs consisting of clustered 5 nm crystallites showing the third harmonics employing magnetic particle spectroscopy (MPS) two times better than Resovist. Alternative synthesis routes of multicore magnetic nanoparticles with a variety of coating possibilities have been reviewed by Gutiérrez et al. [21]. Nevertheless, the water-based syntheses seem to produce the particles with MPI performance improvements below or comparable to the Resovist separated by a size fractionation [12].

Organic-phase iron oxide synthesis generally allows excellent control over particle shape, size, and morphology, and is typically used to produce single-core nanoparticles. Thermal decomposition of an iron (III) oleate precursor, reported by Ferguson et al. [22], yielded 26 nm iron oxide nanoparticles. Stability was achieved through coating with poly(maleic anhydride-alt-1-octadecene)-poly(ethylene glycol) (PMAO–PEG). Such a formulation generated up to three times greater signal intensity than Resovist in MPI [23]. Starmans et al. [24] presented studies on the thermal decomposition of iron oxide hydroxide FeO(OH) resulting in monodisperse 25 nm iron oxide nanoparticles, followed by the encapsulation of magnetic cores in phospholipidic micelles. The particles generated a signal four to six times higher than Resovist at frequencies below 500 kHz. Published experimental data fluctuate around 25 nm as a favorable core size for the current MPI applications utilizing an excitation field frequency of 25 kHz.

Despite a significant effort on the synthesis, multiple independent research groups working on improving iron-oxide-based MPI tracers have all converged on improvements in the order of two to six times better than Resovist.

Nature gives us an example of biological magnetosomes consisting of large iron oxide particles which fulfill the requirements for high MPS amplitudes, having a high saturation magnetization and a low anisotropy constant. Monocrystalline nanoparticles close to 25.7 nm exhibit the third harmonics with a seven-fold increase in comparison to Resovist, the highest reported values so far [25].

All the experimental values are still far below the initial calculations based on the Langevin theory, which predicts a cubic improvement of spatial resolution with iron oxide core diameter; hence, calculating a much higher potential for the improvement of the tracer, suggesting a signal increase even by two orders of magnitude for the core size of 40 nm [1]. The Langevin theory predicts the MPI signal in relation to the particle core size and does not account for the anisotropy of the particle core; thus, in practice, the expected MPI signal would differ [18]. More importantly, Tay et al. [26] show that increasing the magnetic relaxation with core size eventually opposes the expected Langevin behavior, and the high spatial resolution of MPI cannot be further achieved by increasing core size above 25 nm.

The motivation behind our studies was the optimization of iron oxide particles for MPI in a cost- and time-efficient manner, utilizing MPS, which is a zero-dimensional MPI scanner, as a screening tool. In this work, we employed a screening technique in both a cheap and simple aqueous synthesis method, as well as a more costly and laborious organic synthesis approach for the preparation of magnetic particles, aiming for a scope of improvement. In the first approach, we optimized large-scale iron oxide water-based synthesis by varying reaction parameters (temperature, stirring speed, flow rate of precursor addition, dextran molecular size, and type of coordinating agents to iron), resulting in stable dextran-coated nanoparticles (MC-SPIONs). The second approach first yielded hydrophobic highly magnetic particles, which have to be functionalized with hydrophilic molecules in order to establish a redispersed aqueous suspension. Here, a biocompatible poly(ethylene glycol) gallic acid (GA-PEG) coating polymer was applied. The particles were subsequently dialyzed in dextran to further enhance their stability (SC-SPIONs). For our purpose, MPS could be employed as a facile screening technique which allowed for the quantification of particles’ MPI performance regardless of synthesis conditions, thus avoiding the need for the time-consuming recording of an MPI system function. The excellent performance of the optimized particles in both synthesis routes was demonstrated in MPI.

## 2. Materials and Methods

### 2.1. Materials

Ferrous chloride tetrahydrate, ferric chloride hexahydrate, sodium hydroxide and formic acid were purchased from Merck (Merck KGaA, Darmstadt, Germany). Dextran 10 (Dex-10, pharmaceutical grade) and dextran 40 were acquired from Pharmacosmos (Pharmacosmos A/S, Holbaek, Denmark) and AppliChem (AppliChem GmbH, Darmstadt, Germany), respectively. Ferric acetylacetonate and zinc acetylacetonate were obtained from Acros Organics (Acros Organics N.V., Geel, Belgium). Oleic acid (Ph. Eur standard, technical grade) and benzyl ether (98%) were obtained from Sigma-Aldrich (Sigma-Aldrich Chemie GmbH, Steinheim) under the Fluka brand. Poly(ethylene glycol) avg. wt. 10,000 g/mol (PEG 10k), gallic acid (97.5–102.5%), *N*,*N*-dimethylformamide (DMF, >99.8%), dicyclohexylcarbodiimide (DCC, 99%), and 4-(dimethylamino)pyridine (DMAP, >99%), chloroform (>99.8%), diethyl ether (anhydrous, >99.7%), and acetone (>99.8%) were all purchased from Sigma-Aldrich. 50 kDa Biotech Grade CE membrane was used from Spectrum Labs (Spectrum Laboratories Inc., Rancho Dominguez, CA, USA). Filter units Millex-HA 0.45 µm and Whatman 0.2 µm were used. Water was purified by a Milli-Q Gradient machine (Merck KGaA, Darmstadt, Germany) equipped with a 0.16 µm filter. All reagents were used without further purification.

### 2.2. Characterization Techniques

The hydrodynamic size of the iron oxide nanoparticles was determined utilizing dynamic light scattering (DLS) with a Malvern Zetasizer Nano Series (Malvern Instruments Ltd., Worcestershire, UK). Crystallite size and morphology were investigated employing JEOL JEM-1011 transmission electron microscope (TEM) (JEOL Inc., Peabody, MA, USA) at an acceleration voltage of 80 kV. Qualitative X-ray Diffraction (XRD) analysis was performed facilitating PANalytical EMPYREAN X-ray diffractometer (Malvern Panalytical GmbH, Kassel, Germany) applying Cu Kα radiation (1.54056 Å). The Fe concentrations were measured by Thermo Scientific ICP-OES iCAP 7600 (Thermo Fisher Scientific, Waltham, USA) with Y as an internal standard. Zn and Fe concentrations were also determined by Agilent Technologies ICP-MS 7900 (Agilent Technologies Inc., Santa Clara, CA, USA), employing Co as an internal standard. Thermogravimetric analysis (TGA) measurements were conducted by Mettler TGA/DSC thermal analyzer (Mettler Toledo, Columbus, OH, USA) in the temperature range 303.15–1273.15 K under oxygen flow of 50 mL/min.

**Figure 1:**
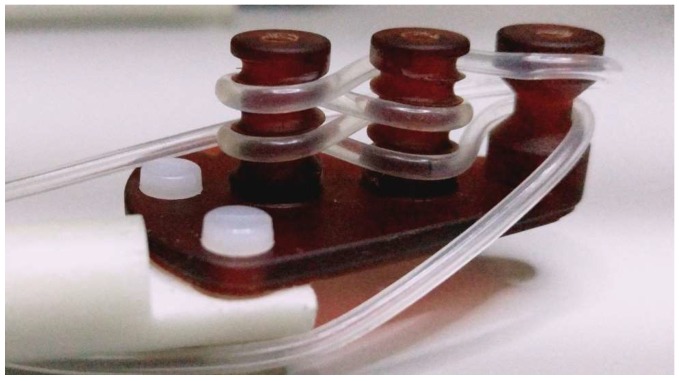
Tube phantom filled with redispersed tracer in water, mounted on the robot arm.

The MPS signal was analyzed employing a commercial magnetic particle spectrometer MPS-3, manufactured by Bruker BioSpin (Ettlingen, Germany), operating at a fixed excitation frequency of 25.25 kHz and an excitation field up to 25 mT. Aqueous samples at different iron concentrations were studied in order to determine the concentration dependency of the odd harmonics. Stationary phantoms were imaged with a preclinical MPI scanner 25/20 FF (Bruker BioSpin/Phillips), installed at Charité University Hospital in Berlin, Germany. This system is a field-free-point (FFP) scanner and employs the system function (SF) approach for image reconstruction [27]. The spatial resolution of MPI is in the range of 1–2 mm and depends on the magnetic properties of a tracer. The temporal resolution of one frame is 21.5 ms, and circa 100 averages were acquired to reduce the noise. A selection gradient field of 2.5 T/m in the *z*-direction (1.25 T/m in *x*- and *y*-directions) is implemented to suppress the response signal of the SPIONs in all regions of the field-of-view (FOV), except for a small volume around the FFP. By moving the FFP through the FOV, a spatially encoded signal of the whole imaging volume can be detected. This movement of the FFP is accomplished by applying three orthogonal drive-fields oscillating at slightly different excitation frequencies about 25 kHz with amplitudes of 12 mT. For the phantom, flexible silicon tubing with an inner diameter of 1.0 mm was used (see Figure 1) and filled with 71 µL iron oxide tracer redispersed in water at concentrations of *c*(Fe) = 5, 1, or 0.5 mM.

Images were captured with a temporal resolution of approx. 1.7 s. The system functions were acquired for a FOV of 26.4 × 26.4 × 13.2 mm^3^ with 33 × 33 × 33 voxels resulting in a voxel size of 0.8 × 0.8 × 0.4 mm^3^. The sample size in the system function recordings was 2 × 2 × 1 mm^3^ (4 µL), with a concentration of 100 mM for Resovist and the MC-SPIONs, and the SC-SPIONs at the original concentration of 50 mM. In the MPI measurements of the phantom, the background was removed from the recorded data. For this purpose, the background signal was recorded at the beginning of the measurement and then the phantom was moved robotically to the center of the FOV. An average rate of 100 is applied, and images of all tracers were reconstructed by selecting 2209 frequency components and applying the Kaczmarz algorithm [28] with 5 iterations and a regularization factor of 1. The reconstructed volumes were rendered with a threshold of 0.4 related to the maximum intensity of the image.

### 2.3. Water-Based Sythesis of Multicore Superparamagnetic Iron Oxide Nanoparticles (MC-SPIONs)

887 mL of ferrous chloride (192.9 g/L), 579 mL of ferric chloride (378.4 g/L), 6480 mL dextran solution (40,000 g/mol, 25 wt %), 218 g of formic acid (98 wt %), and 2054 mL of MilliQ water were introduced into the reactor (30 L Reactor CA 03) while stirring. The mixture was degassed under nitrogen with a flow rate of 100 L/h and heated to 293.15 K. The temperature was maintained at 293.15 K until a completion of the reaction. 9450 mL of NaOH solution (50 g/L) was added within 5 min. The injection was performed between the two lower levels of the Intermig stirrer. The mixture was stirred at 1400 rpm for an additional 120 min with a nitrogen flow of 100 L/h. In the screening experiments, optimizing the synthesis conditions for the following parameters was examined: temperature, stirring speed, flow rate of precursor addition, dextran molecular size, and type of coordinating agent to iron.

### 2.4. Organic Synthesis of Coated Single-Core Superparamagnetic Iron Oxide Nanoparticles Doped with Zinc (SC-SPIONs)

#### 2.4.1. Organic Synthesis of Nanoparticles

The synthesis was performed based on the reaction of iron oxide nanocubes described by Noh et al. [29]. 0.180 g of ferric acetylacetonate and 0.053 g of zinc acetylacetonate were combined in a three-neck 100 mL RBF with 1.2 mL of oleic acid and 10 mL of benzyl ether. The mixture was stirred at 420 rpm. Nitrogen was vigorously bubbled through the solution for 20 min, then the needle was removed from the reaction solution and the flow reduced to a trickle. The flask was inserted into a preheated mantle and the temperature rose to 571.15 K at an average rate of 313.15 K/min. The reaction was maintained at the reflux temperature for 30 min, and then cooled to room temperature. The particles were precipitated using 2:1 ethanol by volume and centrifuged at 3220 rcf for 30 min. The supernatant was discarded. The particles were rinsed twice with ethanol and stored for further use. In the screening experiments aiming at the optimization of the nanoparticles, the following parameters were investigated: zinc concentration, oleic acid content, stirring speed, anhydrous conditions, temperature, and reaction duration. 

#### 2.4.2. Gallic Acid–PEG Ligand Synthesis

Using a DCC-mediated coupling, one molecule of PEG 10k was coupled with one molecule of gallic acid in a modified synthesis based on previously published work [30,31]. In brief, 100 g of PEG 10k, 1.7 g of gallic acid, and 0.3 g of DMAP were combined in a one-neck 2 L round bottom flask in 1 L of DMF under gentle heating and nitrogen flow. The reaction was then stirred vigorously at room temperature while 2.48 g of DCC dissolved in 100 mL of DMF was added dropwise over one hour. Once addition was completed, the reaction was stirred for an additional 3 h.

Following the completion of the reaction, the solvent was removed by vacuum and the product was dissolved in 1 L of acetone with heat and vigorous shaking. The acetone was cooled in an ice-bath to precipitate dicyclohexylurea by-product, which was removed by filtration. The remaining acetone was concentrated to 100 mL and poured into 1 L of cold stirred diethyl ether. The precipitated white product was then isolated by a vacuum filtration, and after that dissolved into chloroform followed by a subsequent precipitation. The off-white solid was dried under high vacuum for 24 h. The yield was determined at approx. 85% *w*/*w*.

#### 2.4.3. Phase Transfer and Purification of Particles

In a typical phase transfer, 50 mg of dried nanoparticles were added to 6.4 mL CHCl_3_ and sonicated for one minute to disperse aggregates. 3.2 mL 0.0625 M GA-PEG ligand in chloroform and 1.6 mL of triethylamine were mixed and shaken vigorously for 12–15 h in a ligand exchange procedure. After shaking, the solution was transferred into a round-bottom flask and MilliQ water was added at a volume twice that of the coating solution. The chloroform was then slowly removed at 100 mbar while vigorously mixing the solution. This resulted in a homogenous aqueous solution of nanoparticles at pH 12. Following the phase transfer, the solution was placed into a 50 kDa dialysis membrane and dialyzed against dextran 10 (12 g/L) for 7 days and stored at 278.15 K until further use.

## 3. Results and Discussion

### 3.1. Nanoparticles Synthesis

MC- and SC-SPIONs were optimized via water-based and organic synthesis, respectively. The optimization procedure was performed by means of MPS; the straightforward technique based on the same physical principle as MPI waiving of any spatial encoding [32]. Only homogeneously redispersed samples in water were selected for the MPS screening.

**Figure 2:**
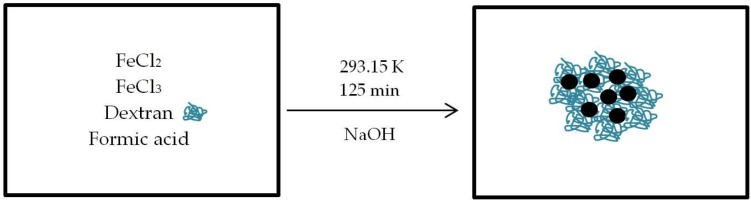
Schematic overview of multicore superparamagnetic iron oxide nanoparticle (MC-SPION) synthesis in aqueous media.

The water-based synthesis of MC-SPIONs (schematic overview in Figure 2) was performed directly in a 30 L reactor, offering high-level control over reaction parameters. The well-established method of chemical coprecipitation from ferrous and ferric chloride in basic conditions was explored. Various carboxylic acids were studied as potential coordinating agents for iron. Formic acid was found to have the most positive impact on the crystal growth and phase purity. It was established that dextran had to be added at the beginning of the reaction in order to realize the optimal precipitation temperature. Dextran with MW 40,000 g/mol yielded the best combination of particle stability and magnetic properties. Temperatures in the range of 293.15 to 333.15 K were studied, but no MPS improvement was found with a temperature change. Sodium hydroxide addition was evaluated in terms of agglomeration homogeneity. The best control over particle size and distribution was accomplished with the base added between the two lower levels of the Intermig stirrer and additional duration of 5 min. The optimized product is colloidally stable for years at room temperature, and shows consistent MPS performance across multiple dilutions. The maghemite/magnetite phase was determined by X-ray Diffraction (XRD) analysis. The brown color of the product suggests the presence of maghemite rather than magnetite.

**Figure 3:**
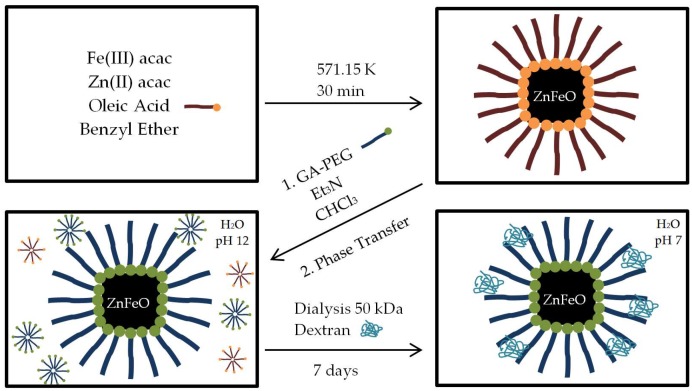
Schematic overview of single-core (SC)-SPIONs synthesis in organic media, subsequent PEG functionalization and a phase transfer to water, followed by a dialysis in a dextran solution.

The organic synthesis of SC-SPIONs was conducted by a thermal decomposition of ferric acetylacetonate in benzyl ether [33]. A schematic overview, including a surface functionalization and a phase transfer to water, followed by a dialysis, is displayed in Figure 3.

The synthesis process was tailored to provide particles large enough for superior MPS performance, but at the same time still stable for months in aqueous media. A cubic shape was aimed for, expecting a higher magnetisation value than for spheres resulting from a smaller surface anisotropy [29]. In addition, it was decided to dope with zinc in order to enhance magnetic properties. According to theoretical considerations, the net magnetic moment of ferrite per unit cell is determined by the magnetic moment of divalent ions, while the magnetic spins at the octahedral sites are parallel to each other and antiparallel to the spins at tetrahedral sites. Zinc, which has a magnetic moment of zero, has a preference to occupy tetrahedral sites, leading to a decrease in the amount of antiparallel interactions and an increase in magnetism [34]. Here, the optimal zinc content in the synthesis was based on empirical measures by MPS. After thorough screening, the best-performing synthesis utilized an iron/zinc molar ratio of 2.5, which resulted in a ratio of 16.3 in the particles.

It was found that the degassing step under vacuum typically led to a more monodisperse particle distribution after 30 min of heating, but with a much smaller particle size than expected. Additional heating to achieve an acceptable size would bring the size distribution back to the level of the regular synthesis. A vigorous nitrogen bubbling instead of the degassing step under vacuum, the use of anhydrous reagents, and the high-temperature heating of glassware prior to use were implemented to prevent water from entering the reaction system.

In order to transfer the SC-SPIONs into aqueous media, a polyethylene glycol polymer was utilized. PEG is one of the most thoroughly investigated polymers for nanoparticle encapsulation, owing to a favorable biocompatibility profile, the ability to increase blood circulation time, and high solubility in aqueous media [35]. In addition to this, polyethylene glycols are inexpensive, offer functional groups for further functionalization, are linear, and readily available in a variety of sizes, facilitating the ability to tune the thickness of the particle coating in order to optimize magnetic properties.

PEG 10,000 provides a coating thick enough to prevent interactions with neighboring particles, but still sufficient to preserve the Brownian motion of the particles. The linking group, gallic acid, has a high tolerability (a median lethal dose administered intravenously LD50 i.v. is 320 mg/kg in mice), and has been shown to have a high affinity for metal oxides [36]. The ideal scenario would be a complete exchange of the oleic acid carboxyl groups with the trihydroxyl groups of the gallic acid linker. A study of such a ligand exchange, using 3,4-dihydroxy-phenylalanine functionalized PEG, indicated a substantial removal of oleic functionalities from the particle surface [37]. We found that compared to conventional catechol linkers, using gallic acid–PEG resulted in fewer large aggregates during the phase transfer step and increased stability after the addition of dextran. It has been suggested that gallic acid coordinates via two phenoxy groups, with the third remaining unattached [36]. This may lead to a more irregular packing of PEG chains on the nanoparticles, facilitating the incorporation of dextran into the coating matrix.

### 3.2. Particle Size

**Figure 4:**
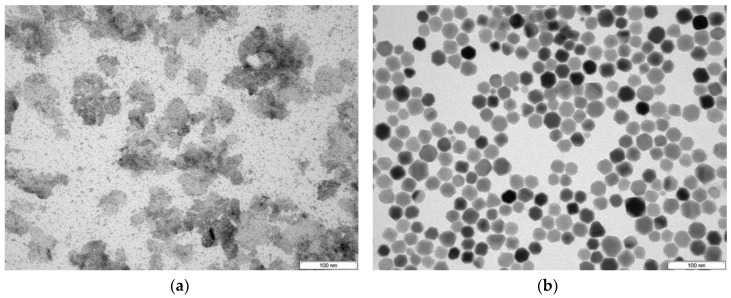
Transmission electron microscope (TEM) images of (**a**) MC-SPIONs and (**b**) SC-SPIONs, with a 100-nm scale bar.

The TEM images shown in Figure 4 depict the differences in the particle shape and size in the two studied approaches. MC-SPIONs obtained by aqueous synthesis consist of small nanoparticles in the 5-nm region (Figure 4a). Dextran appears to form patches of iron oxide-containing agglomerates. In contrast, Figure 4b displays well-redispersed SC-SPIONs of 27 ± 5 nm. The particles tend to be of a hexagonal and cubic shape, which are coated with a very thin shell having a spherical shape. The particle size is within the range of ideal size for MPI, as argued by the research community [22,25]. It seems that dextran is well-incorporated into the coating matrix. The large dextran polymers were intended to function as spacer groups and prevent the formation of particle aggregates over long storage times. This modification allowed the production of a highly stable PEG dextran SC-SPIONs suspension.

**Figure 5:**
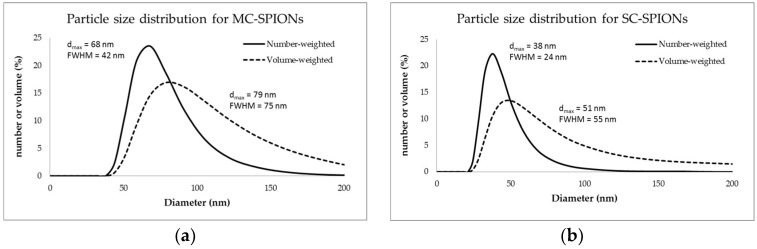
Hydrodynamic particle diameter distribution weighted by number (solid lines) and volume (dashed lines) measured by dynamic light scattering (DLS) for (**a**) MC-SPIONs and (**b**) SC-SPIONs. d_max_ is the diameter corresponding to the maximum of the peak. FWHM stands for the full width at half maximum of the peak.

The DLS hydrodynamic diameter (Figure 5b) for SC-SPIONs was 38 nm, as determined by number-weighted analysis, which represents the iron core plus the coating consisting of PEG and dextran. The dominant peak was at 68 nm for MC-SPIONs (Figure 5a), indicating multiple iron oxide cores embedded in dextran. Hydrodynamic particle size distribution in the volume-weighted analysis is shifted towards higher numbers; 51 nm and 79 nm for SC-SPIONs and MC-SPIONs, respectively. The peak for SC-SPIONs has an extended right shoulder, indicative of a small fraction of aggregates. The average hydrodynamic diameter (*Z*_av_) was determined to be larger for both studied samples; 96 nm (Polydispersity Index (PDI) 0.24) and 104 nm (PDI 0.11) for SC-SPIONs and MC-SPIONs, respectively, compared to 62 nm for Resovist [38].

### 3.3. MPS Analysis and MPI Imaging

**Figure 6:**
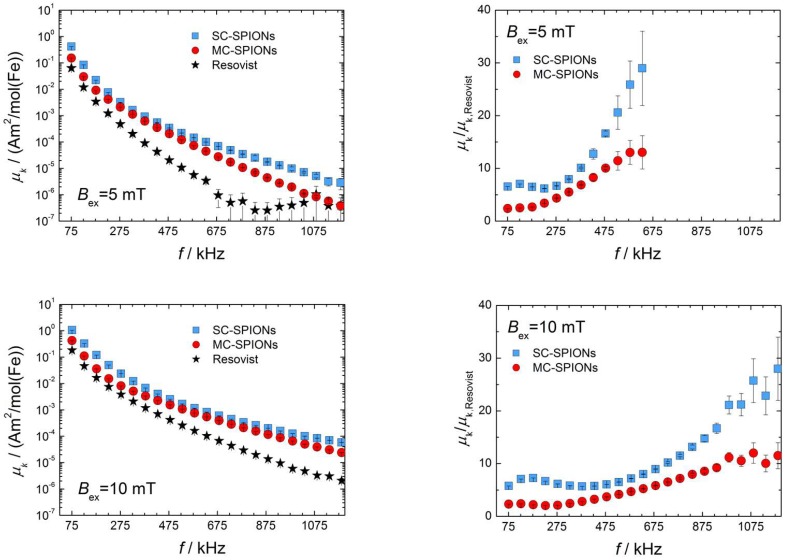

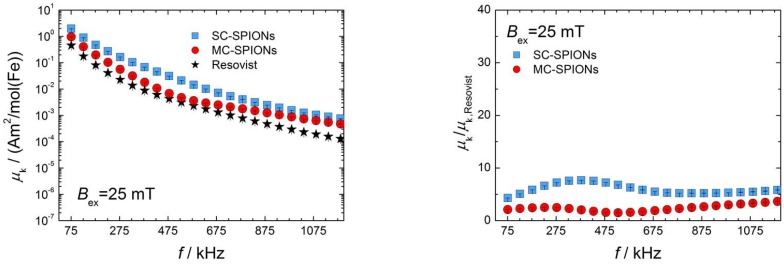
Magnetic particle spectroscopy (MPS) experimental data (odd harmonics only) of SC-SPIONs, MC-SPIONs and Resovist plotted as magnetic moment *µ_k_* versus frequency *f* (**left column**); and magnetic moment ratios of SC-SPIONs and MC-SPIONs versus Resovist defined as *µ_k_/µ_k_,_Resovist_* (**right column**) at an excitation field *B_ex_* of 5 mT, 10 mT, and 25 mT. Data points are shown up to a relative uncertainty of 50%.

The MPI performance was routinely investigated by MPS at an excitation frequency of 25.25 kHz. The MPS response in all cases was tested at pH 6–8 and across a range of iron concentrations from 1 mM to 50 mM, to confirm the independence of tracer performance from particle concentration (as estimated by the iron concentration). The full curves for MPS data at 5 mT, 10 mT, and 25 mT are displayed in Figure 6 (left column). Data points are presented only to a relative uncertainty of 50%. An increase in the magnitude of the harmonics with increasing field strength coincides with theoretical models [39]. Improvement factors obtained at excitation amplitudes of 5 mT, 10 mT, and 25 mT are presented up to 1200 kHz in Figure 6 (right column). As can be seen, the improvement factors increase with decreasing field strengths.

The ratios of the amplitudes of the third harmonic against Resovist were determined at 2.4, 2.3, and 2.1 for MC-SPIONs at 5 mT, 10 mT, and 25 mT, respectively. We can speculate that such MPS enhancement has its roots in a larger average hydrodynamic size of MC-SPIONs (104 nm), in comparison to Resovist (62 nm). Although the structure of Resovist is very complex, as revealed by the finding of two populations of sizes [10], the presence of the larger population is of key importance for the good MPI performance of Resovist. This has been demonstrated by the fractionation of Resovist [12], which results in an MPS signal change of up to 2.2 for the isolated larger clusters. Also, another direct synthesis of multicore iron oxide nanoparticles [20] led to an increase in the MPS amplitude by a factor of two for Nanomag-MIP particles with a hydrodynamic diameter of 106 nm.

**Figure A1:**
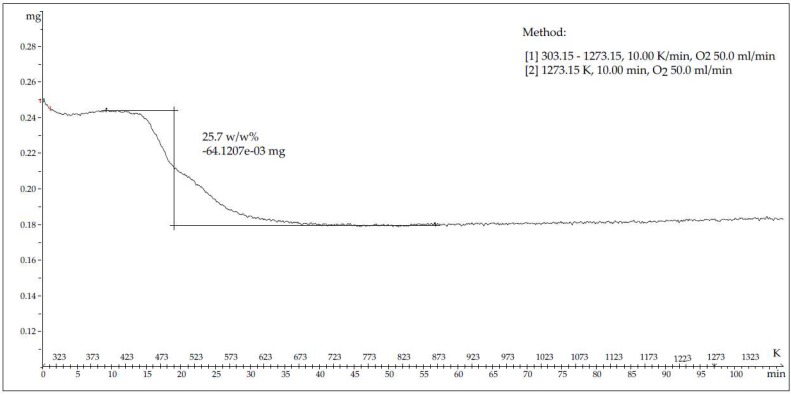
TGA analysis of a dried sample of SC-SPIONs with GA-PEG ligands before dialysis in dextran. The sample was heated up under oxygen atmosphere at 10 K/min and the weight loss was recorded as a function of temperature. The remaining mass of inorganic material was 74.3%.

The MPS signal improvement at the third harmonic for SC-SPIONs was found to be 6.6, 5.8, and 4.3 at 5 mT, 10 mT, and 25 mT, respectively. In comparison, single-core UW-2 nanoparticles with a core size of 27 nm and hydrodynamic diameter of 47 nm showed improvement of the third harmonic intensity of only 3.5-fold [22]. Iron oxide nanoparticles encapsulated in lipidic micelles possessing a core diameter of 25 nm and hydrodynamic diameter of 61 nm generated four to six times more signal than Resovist at frequencies below 0.5 MHz [24]. When comparing our SC-SPIONs to these published approaches, it appears that the nanoparticles presented here have a similar size (by TEM), but contain zinc as a dopant known to increase particle magnetism; possess a thin layer of GA-PEG coating (based on TEM, see Figure 4b, and TGA analysis, see Figure A1 in Appendix A); and are additionally stabilized with dextran, resulting in an average hydrodynamic particle size of 96 nm, leading to an increased colloidal stability which can have an impact on the MPS signal.

**Figure 7:**
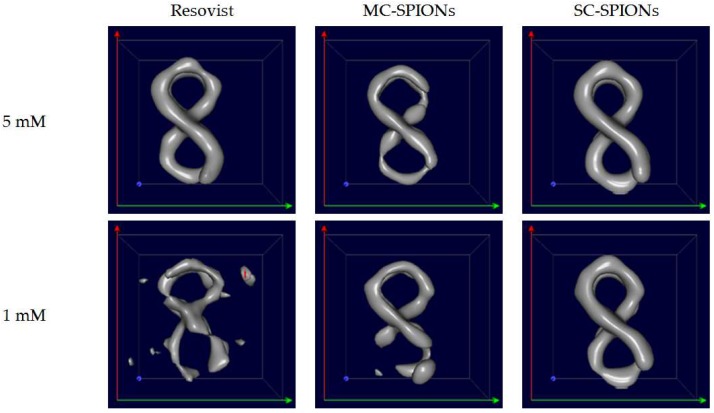

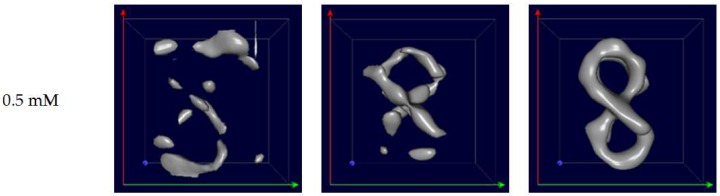
Reconstructed MPI images of phantoms using 5 mM, 1 mM, and 0.5 mM of Resovist, SC-SPIONs, and MC-SPIONs.

The superior MPS performance of the purposely optimized tracers was confirmed on a preclinical MPI scanner (Bruker BioSpin GmbH, Ettlingen, Germany). Accurately reconstructed images visualizing the geometric shape of the tube phantom containing 71 µL of iron oxide particles were obtained at the concentration of 5 mM of iron for all three investigated tracers (Figure 7), which set a sensitivity baseline for Resovist. A further decrease in the iron concentration to 1 mM led to the loss of a full phantom reconstruction for Resovist. MC-SPIONs resulted in image reconstruction superior to Resovist. However, as predicted by MPS performance, SC-SPIONs outperformed both MC-SPIONs and Resovist. Lowering the iron concentration to 0.5 mM led to a full phantom reconstruction for SC-SPIONs only. Nevertheless, the further reduction of the tracer concentration is required for clinical application. Due to a lack of standardization of MPI acquisition, data from different systems are not directly comparable. 

## 4. Conclusions

We demonstrated that the MPS-driven screening of synthesis methods is a facile technique for selecting optimized MPI tracers. Improved MPI performance is already shown in the amplitude of the third harmonic of the MPS, which requires only seconds to be recorded, in comparison to the several hours spent on acquiring system function and then image reconstruction in MPI. Although MPS is a zero-dimensional magnetic particle imaging system, it provides a robust set of characterization data of imaging properties, which can be easily compared between various iron oxide nanoparticles formulations once the same reference standard (e.g., Resovist) is utilized.

MPS enhancement of up to 2.5-fold was determined for MC-SPIONs obtained via coprecipitation in aqueous media in a large-scale process. A reconstructed MPI image of a tube phantom demonstrates this improvement. An organic synthesis method generates well-defined SC-SPIONs with even higher MPS signal intensities, which in consequence leads to a 6.6-fold signal increase at the third harmonics at 5 T compared to commercially available Resovist. The superior MPI performance of SC-SPIONs estimated by MPS was reflected by MPI experiments visualizing the geometric shape of the phantom at iron concentrations as low as 0.5 mM.
